# Who experiences discrimination in Brazil? Evidence from a large metropolitan region

**DOI:** 10.1186/1475-9276-11-80

**Published:** 2012-12-18

**Authors:** James Macinko, Pricila Mullachery, Fernando A Proietti, Maria Fernanda Lima-Costa

**Affiliations:** 1 Dept. of Nutrition, Food Studies & Public Health, New York University, New York, USA; 2 Dept. of Social and Preventive Medicine, Federal University of Minas Gerais, Belo Horizonte, Brazil; 3 René Rachou Research Institute, Oswaldo Cruz Foundation, Belo Horizonte, Minas Gerais, Brazil

**Keywords:** Discrimination, Brazil

## Abstract

**Introduction:**

Perceived discrimination is related to poor health and has been offered as one explanation for the persistence of health inequalities in some societies. In this study, we explore the prevalence and correlates of perceived discrimination in a large, multiracial Brazilian metropolitan area.

**Methods:**

The study uses secondary analysis of a regionally representative household survey conducted in 2010 (n=12,213). Bivariate analyses and multiple logistic regression assess the magnitude and statistical significance of covariates associated with reports of any discrimination and with discrimination in specific settings, including when seeking healthcare services, in the work environment, in the family, in social occasions among friends or in public places, or in other situations.

**Results:**

Nearly 9% of the sample reported some type of discrimination. In multivariable models, reports of any discrimination were higher among people who identify as black versus white (OR 1.91), higher (OR 1.21) among women than men, higher (OR 1.33) among people in their 30’s and lower (OR 0.63) among older individuals. People with many health problems (OR 4.97) were more likely to report discrimination than those with few health problems. Subjective social status (OR 1.23) and low social trust (OR 1.27) were additional associated factors. Perceived discrimination experienced while seeking healthcare differed from all other types of discrimination, in that it was not associated with skin color, social status or trust, but was associated with sex, poverty, and poor health.

**Conclusions:**

There appear to be multiple factors associated with perceived discrimination in this population that may affect health. Policies and programs aimed at reducing discrimination in Brazil will likely need to address this wider set of interrelated risk factors across different populations.

## Introduction

Experiences of discrimination, defined as manifestation of negative attitudes, judgments, or differential treatment on the basis of gender, race, social class, or other characteristics that disadvantages a social group,[1] have emerged as an important cause of poor health and one explanation for the persistence of health inequalities in some societies [2-7]. Discrimination is believed to contribute to poor mental and physical health through several pathways, including structural factors such as differential rates of arrest and incarceration, disparities in wages, unequal educational and housing opportunities, and unequal access to and treatment in social services [3,5,8,9]. Additional evidence points to biological mechanisms linking discrimination with cumulative exposure to stress and subsequent deterioration of mental and physical health status and the emergence of additional risk factors [10-12]. Relationships between perceived discrimination and health have also been identified through differences in access to and use of health services, and through coping strategies that may result in less than optimal health behaviors [13-17].

Much of the existing literature–often focusing on discrimination based on the victim’s perceived race or skin color–comes from the United States [12]. Research points to a set of underlying mechanisms that may link experiencing discrimination with health inequalities, but there is much to be learned about how different risk factors for experiencing discrimination may interrelate and how they may differ in different settings [7,15,16,18]. For these reasons, cross-national studies on discrimination and health can be helpful in understanding how and where discrimination occurs, identifying risk and protective factors, and developing mechanisms to mitigate the negative impacts of discrimination on health.

The study of discrimination in Brazil may be particularly fruitful, given the country’s large, multi-racial population, complex race and social relations, and considerable social and economic diversity. In Brazil, studies on perceived discrimination point to the importance of key demographic characteristics such as skin color, socio-economic status (SES), and gender. Existing studies suggest that people who describe their skin color as other than white, women, less educated individuals, and sexual minorities are more likely to report having experienced some sort of discrimination [19-22]. The literature has also documented differences in access to health care and quality of care received by different underprivileged groups in society. For example, black and poor women have reduced access to antenatal care and low quality postnatal care [14,16]. Other studies revealed significant differences in coverage of clinical breast examination based on SES and skin color [15]. In terms of health outcomes, Brazilian studies have found positive correlations between self-reported discrimination and poor self-rated health as well as adverse health behaviors [19,23-25]. In a nationally-representative survey, nearly one tenth of recent users of outpatient health services reported feeling that they had been victims of some type of discrimination [20].

This paper contributes to the discussion of discrimination and health by exploring the prevalence and correlates of self-reported discrimination, based on a recent representative sample of a large metropolitan region in Brazil. Our objective is to gain better understanding of the distribution, relative magnitude, and interrelationships among risk factors for exposure to discrimination within a large, multiracial, metropolitan area.

## Methods

The study took place in the Belo Horizonte Metropolitan Region, which includes the city of Belo Horizonte (the capital of the state of Minas Gerais and home to 2.4 million inhabitants) and about 2 dozen surrounding municipalities. The total population in the region is nearly 5 million inhabitants [26]. Although the city has Brazil’s fourth largest GDP and a high Human Development index (0.84), it has large inequalities in health and socioeconomic conditions [27].

We use data from the second Household Health Survey of the Metropolitan Region of Belo Horizonte conducted in July 2010. Data collection was carried out via a supplement to the Regional Employment and Unemployment Survey, carried out by the João Pinheiro Foundation—an organization associated with the state government of Minas Gerais [28]. This survey, based on a sample of 7,500 households with about 24,000 respondents, is undertaken on a regular basis and is designed to be representative of the non-institutionalized population residing in the Belo Horizonte Metropolitan Region. The sampling design is two staged: strata are identified at the census tract level and households within census tracts are the primary sampling unit. More details on the survey design can be found in prior publications and online [29-31].

Of the total households selected, 5,802 (77.3%) participated in the survey. All residents in the sampled households over the age of 20 were eligible to participate in the health supplement via face to face interview, making a total of 12,979 participants [27,28]. The health supplement was reviewed and approved by the IRB of the *Instituto de Pesquisas René Rachou da Fundação Oswaldo Cruz* in Belo Horizonte, Minas Gerais.

Our dependent variable was the respondents’ self-report to the question, “Have you felt yourself to be the victim of any type of discrimination?” Respondents were then read a list of options, including “when seeking healthcare services, in the work environment, in the family, in social occasions among friends or in public places, and/or in other situations”. An indicator variable, termed “any discrimination” was developed to capture whether the respondent replied yes to any of these sub-categories. This measure was used because of its sensitivity, that is, its ability to capture the widest experience of different types of perceived discrimination [32]. We used this broad measure because our objective was to obtain an indication of the prevalence of multiple forms of discrimination and associated factors in the Belo Horizonte metropolitan region.

The main exposure variables included the respondents’ self-reported skin color, based on those in the Brazilian census categories that include: white (branca), black (preta), brown (parda), and yellow (amarela). The latter category (amarela) had only 11 respondents and was dropped from analyses. There were no respondents who identified themselves as “indigenous”, although 38 individuals had missing values for skin color and were also dropped from analyses. Other exposure variables were gender (male/female), and a set of measures of socioeconomic status including education (categorized into less than 4 years, 4–7 years, 8–12 years, and 12 or more years and which correspond to less than primary school, completed primary school, completed high school, and some college) and household wealth. We use household wealth as a proxy for income to better capture extremes on both ends of the income distribution. To do this, we created a household wealth index by performing principal component analysis on a list of 12 household goods. The analysis resulted in only one factor with an Eigenvalue over 1, so this factor was extracted and the resulting score was divided into quintiles [33,34]. We also measured individual’s sense of relative social position using the MacArthur scale of subjective social status [35].

Other descriptive variables include age and a composite measure of poor health status. Because of collinearity among health indicators, we created a composite measure based on principal component analysis of reports of poor/very poor self-rated health, number of the last 30 days spent in poor mental and/or physical health, and history of medical diagnosis of one or more of a list of 8 common chronic conditions. Only one factor had an Eigenvalue over 1, so it was extracted. Given its distribution, this factor, which we term “poor health score”, was divided into tertiles. The lowest tertile, representing “few health problems”, is used as the reference group.

Two contextual variables were also analyzed. The first captures whether the person resides in the city of Belo Horizonte or one of its surrounding municipalities. The second, termed “social trust” is used to capture aspects of the individual’s social environment. It is composed of respondents’ answers to whether “most people can be trusted” and whether “most people would take advantage of you if they could”. The variable is coded as 1 (i.e. the respondent exhibits low social trust) if the person responded negatively to the first question and positively to the second.

Bivariate analyses of differences in proportions were based on design-corrected Pearson (F-tests) between the population proportion and each sub-group. Multivariable analyses use logistic regression (since the main outcome is relatively infrequent) to assess the magnitude and statistical significance of a set of correlates thought to be associated with experience of discrimination. Results are presented as a series of nested models to illustrate the effect of adding additional sets of variables to the models. All main explanatory variables were tested for interactions. Only one set of interaction terms was statistically significant (skin color and residence in Belo Horizonte) and was included in the final analyses. Based on results of the full models, adjusted probabilities were calculated using the Stata statistical package version 12’s “margins” command [36]. Results are displayed graphically to visualize different combinations of variables. Adjusted probabilities are calculated based on the previously fitted full multivariable model, by setting a variable of interest at a specific value and then calculating the values of the outcome variable across a specified range, while all other covariates are held at their mean [36]. All analyses (including predicted probabilities) control for the complex sample using Stata’s “svy” commands. Standard errors are corrected for the two stage sample, first by stratum (census tract), then by primary sampling unit (household) and incorporate sample weights. Adjustment of standard errors for household clustering is particularly important given that all adults aged 20 years and over in the selected households were interviewed and these individuals (the mean number of individuals interviewed per household was 2.2 and varied from 1 to 8) are likely to be more similar to each other than to people from other households [37]. Full models were tested for collinearity, which was found to be very low (the average Variance Inflation Factor was <1.5).

## Results

Table 1 shows the distribution of all variables by self-reported skin color. Nearly 49% identified as white, 43% as *pardo* or brown, and 8% as black. There were few differences in sex or age according to skin color group. Those in the white group were less likely to report less than 4 years of schooling and more likely to report having 12 or more years than the general population. Those in the black group had nearly double the population average of those with under 4 years and about 60% fewer adults with 12 or more years of education than the general population. The pardo group had larger numbers of individuals in the middle of the educational distribution. The white group had fewer members in the lowest wealth quintile, while the black group had the highest, followed by the pardo group. In terms of health status, the black group had the lowest proportion of those in the category with the fewest health problems and the pardo group had higher than average proportion in that group.

**Table 1:** Descriptive statistics, by self-reported skin color (%)

	**White**^**1**^	**Black**	**Pardo (brown)**	**Total**
N (unweighted)	6,527	1,121	5,282	12,930
% (weighted)	48.86	8.35	42.78	100.00
Aged 20–29 years	23.76	24.56	26.80	25.13
30-39 years	21.11	21.49	22.99	21.95
40-49 years	19.75	19.16	20.26	19.92
50-59 years	15.78	16.49	15.79	15.84
60+ years	19.60	18.30	14.16*	17.17
Male	45.58	43.75	47.71	46.34
Female	54.42	56.25	52.29	53.66
Schooling (<4 years)	6.35*	16.34*	9.42	8.5
4-7 years	18.22*	31.66*	27.72*	23.41
8-12 years	44.61	43.75	50.6*	47.10
12+ years	30.82*	8.24*	12.26*	20.99
> lowest income quintile	86.75*	69.67*	76.05*	80.75
lowest income quintile (poor)	13.25*	30.33*	23.95*	19.25
Few health problems	61.72	57.53*	66.99*	63.63
Some health problems	25.94	28.59	22.58	24.72
Many health problems	12.34	13.88	10.43	11.65
Highest social position (top 2 tertiles)	66.77	52.20*	62.56	63.75
Lowest social position (lowest tertile)	35.30	47.80*	37.44	36.25
Highest social trust (top 2 categories)	80.88	85.39	85.37	83.18
Lowest social trust (versus top 2 categories)	19.12	14.61	14.63	16.82
Non BH city resident	35.37*	48.76*	44.72*	40.49
BH city resident	64.63*	51.24*	55.28*	59.51
No report of discrimination	91.60	84.85*	92.44	91.39
Reported discrimination (any)	8.40	15.15*	7.56	8.61
In work place	3.32	6.50*	3.00	3.45
In seeking medical care	1.99	2.18	2.05	2.03
In the family	1.40	2.68*	1.24	1.44
In social occasions	4.21	9.71*	3.38	4.32
In other situations	2.17	4.73*	2.20	2.40

Numbers are design-corrected weighted proportions.

Note that “amarelo” and “unknown” skin color categories dropped due to low prevalence (<1%).

*= significant difference from “total” category (p<0.05).

Subjective social status was lowest in the black group, while the white and pardo groups were not different from the population average. There were no differences in levels of social trust among the groups. Whites were more likely and black and pardo respondents less likely to live within the city of Belo Horizonte.

Reports of discrimination also varied by group, with the highest report (15%) among those who identified as black, nearly double the population average. Members of this group also reported higher instances of each specific type of discrimination than the average, except for discrimination in seeking healthcare, which did not differ by skin color group.

Table 2 presents results of multiple logistic regression of report of any type of discrimination. Those who identify as black have a consistently higher odds of reporting discrimination--nearly twice that of their white counterparts. Women are also consistently more likely to report discrimination than men. Compared to those in their 20s, people in their 30s are more likely to report experiences of discrimination. Once health status is included in the model, people in their 60’s or older were less likely to report discrimination than those in their 20’s.

**Table 2:** Self-report of ANY form of discrimination (n=12,213)

	**Model 1**	**Model 2**	**Model 3**	**Model 4**	**Model 5**	**Model 6**	**Model 7**	**Model 8**	**Model 9**
Black skin color	1.95***	1.94***	1.93***	1.9***	1.89***	1.93***	1.91***	1.91***	1.55*
(versus white)	1.53,2.48	1.53,2.48	1.52,2.47	1.48,2.44	1.46,2.45	1.48,2.53	1.46,2.50	1.46,2.50	1.10,2.18
Brown skin color	0.89	0.89	0.89	0.88	0.88	0.93	0.94	0.94	0.71*
(versus white)	0.76,1.05	0.75,1.05	0.76,1.05	0.75,1.04	0.74,1.04	0.79,1.11	0.79,1.11	0.79,1.11	0.54,0.93
30-39 years old		1.4**	1.39**	1.39**	1.4**	1.3*	1.31*	1.31*	1.33*
(versus 20–29 years)		1.13,1.74	1.12,1.73	1.12,1.73	1.12,1.74	1.05,1.62	1.05,1.64	1.05,1.63	1.07,1.66
40-49		1.42**	1.41**	1.42**	1.42**	1.16	1.18	1.18	1.19
(versus 20–29 years)		1.15,1.76	1.14,1.75	1.15,1.75	1.15,1.77	0.94,1.45	0.95,1.46	0.95,1.46	0.95,1.47
50-59		1.34**	1.32*	1.33*	1.34*	0.93	0.94	0.94	0.96
(versus 20–29 years)		1.07,1.67	1.06,1.65	1.07,1.66	1.06,1.69	0.72,1.19	0.74,1.21	0.74,1.21	0.75,1.22
60+		1.1	1.07	1.07	1.05	0.61***	0.62**	0.62**	0.63**
(versus 20–29 years)		0.87,1.40	0.84,1.36	0.84,1.36	0.80,1.38	0.45,0.81	0.46,0.83	0.46,0.83	0.46,0.84
Female			1.31***	1.31***	1.31***	1.22**	1.21**	1.21**	1.21**
(versus male)			1.16,1.49	1.16,1.49	1.15,1.49	1.07,1.39	1.06,1.38	1.06,1.38	1.06,1.38
Poorest wealth quintile				1.11	1.11	1.03	1.01	1.01	1.01
(versus top 4 quintiles)				0.91,1.35	0.90,1.36	0.84,1.28	0.82,1.24	0.82,1.24	0.82,1.25
4-7 years schooling					0.85	0.95	0.96	0.96	0.96
(versus <4 years)					0.65,1.11	0.72,1.25	0.73,1.26	0.73,1.26	0.73,1.27
8-11 years schooling					0.9	1.08	1.12	1.11	1.13
(versus <4 years)					0.68,1.19	0.81,1.44	0.84,1.49	0.84,1.48	0.85,1.49
12+ years schooling					0.9	1.1	1.17	1.17	1.23
(versus <4 years)					0.65,1.24	0.79,1.54	0.84,1.64	0.83,1.64	0.88,1.67
Some health problems						2.29***	2.29***	2.29***	2.27***
(versus few)						1.91,2.75	1.90,2.75	1.90,2.75	1.89,2.72
Many health problems						4.98***	4.94***	4.94***	4.97***
(versus few)						4.08,6.08	4.05,6.03	4.05,6.02	4.07,6.07
Lowest subjective social status							1.23**	1.23**	1.23*
(versus highest 2 tertiles)							1.05,1.45	1.05,1.45	1.05,1.44
Lowest social trust							1.27*	1.27*	1.27*
(versus highest 2 categories)							1.05,1.54	1.05,1.54	1.05,1.54
BH city								1.02	0.79*
(non-BH city dweller)								0.86,1.20	0.63,1.00
BH city* Black									1.79**
(versus non BH, white)									1.21,2.64
BH city* Brown									0.91
(versus non BH, white)									0.72,1.14

Figures are odds ratios and 95% confidence intervals from logistic regression. Results are adjusted for complex sample design and includes sample weights.

*p<0.05; ** p<0.01; ***p<0.001.

Poverty and educational attainment were not associated in any model with discrimination. Those with the lowest subjective social status and those with low social trust were about one-fifth more likely to report discrimination than those with higher levels of these measures. The presence of health problems was consistently associated with higher reports of discrimination. Individuals with the highest levels of health problems had much higher odds of reporting discrimination than those with the fewest health problems. Finally, residence in the city of Belo Horizonte had a negative (protective) relationship for whites, but a positive relationship for blacks.

Table 3 breaks down the discrimination measure into its distinct types as presented in the questionnaire. For each discrimination category, higher odds were reported among people who identify as black, except in the case of discrimination in seeking medical care, which was not significant for any group. The association with age detected in the overall measure of any discrimination was not present in any systematic way. Schooling exhibited little association with any specific form of discrimination. In the case of discrimination experienced while seeking medical care, both women and those in the lowest income quintile report higher odds than their reference categories. The relationship between poor health and discrimination was positive and consistent for each discrimination type and was highest for those in the worst health. Low subjective social status and low social trust were of similar magnitude and positively associated with discrimination, except in the case of medical care and in “other” occasions. Finally, the interaction of residence in Belo Horizonte and black skin color was positive and significant for all categories except for medical care.

**Table 3:** Correlates of TYPE of discrimination reported (n=12,213)

	**In Health services**	**At work**	**In the family**	**In Social occasions**	**In Other situations**
Black skin color	0.71	1.75*	2.41*	1.96***	1.31
(versus white)	0.33,1.55	1.10,2.81	1.16,5.02	1.32,2.92	0.68,2.51
Brown skin color	0.81	0.71	1.03	0.5***	0.75
(versus white)	0.49,1.31	0.48,1.04	0.55,1.96	0.35,0.73	0.46,1.22
30-39 years old	1.45	1.47*	0.92	1.11	1.34
(versus 20–29 years)	0.94,2.23	1.07,2.01	0.57,1.48	0.83,1.50	0.93,1.94
40-49	1.1	1.43*	1.05	1.07	0.88
(versus 20–29 years)	0.70,1.72	1.05,1.96	0.63,1.74	0.80,1.43	0.58,1.34
50-59	1.14	1.13	1.11	0.82	1.01
(versus 20–29 years)	0.71,1.85	0.78,1.64	0.64,1.95	0.58,1.16	0.65,1.59
60+	0.8	0.62*	0.55	0.62*	0.6
(versus 20–29 years)	0.45,1.41	0.39,1.00	0.29,1.03	0.42,0.93	0.34,1.04
Female	1.33*	1.01	1.24	1.01	1.06
(versus male)	1.01,1.76	0.83,1.23	0.91,1.70	0.84,1.21	0.83,1.35
Poorest wealth quintile	1.62*	1.15	1.08	0.89	0.81
(versus top 4 quintiles)	1.08,2.42	0.86,1.52	0.69,1.68	0.66,1.18	0.56,1.18
4-7 years schooling	1.07	0.98	1.22	1.02	1.02
(versus <4 years)	0.67,1.72	0.62,1.56	0.72,2.07	0.70,1.50	0.62,1.68
8-11 years schooling	1.28	1.59	1.16	1.13	1.04
(versus <4 years)	0.76,2.17	0.99,2.56	0.63,2.14	0.75,1.69	0.62,1.74
12+ years schooling	1	1.97*	1.19	1.16	1
(versus <4 years)	0.50,2.00	1.15,3.36	0.55,2.61	0.73,1.85	0.53,1.89
Some health problems	1.56*	2.15***	2.33***	1.95***	1.87***
(versus few)	1.07,2.28	1.63,2.83	1.58,3.44	1.50,2.53	1.31,2.67
Many health problems	4.45***	4.11***	6.24***	4.32***	3.99***
(versus few)	3.01,6.59	3.06,5.52	4.09,9.50	3.32,5.62	2.83,5.64
Lowest social status	1.28	1.43**	1.5*	1.26*	1.32
(versus highest 2 tertiles)	0.98,1.69	1.15,1.79	1.07,2.11	1.01,1.57	0.98,1.79
Lowest social trust	1.1	1.48**	1.55*	1.35*	1.04
(versus highest 2 categories)	0.76,1.58	1.14,1.91	1.04,2.31	1.04,1.76	0.72,1.50
BH city	0.79	0.82	1.58	0.75	0.93
(non-BH city dweller)	0.51,1.22	0.59,1.14	0.91,2.72	0.55,1.03	0.59,1.47
BH city* Black	0.82	1.97*	2.32*	2.12**	2.86***
(versus non BH, white)	0.39,1.75	1.09,3.57	1.07,5.03	1.34,3.37	1.58,5.17
BH city* Brown	0.9	1.02	1.43	0.85	1.19
(versus non BH, white)	0.58,1.38	0.74,1.41	0.79,2.56	0.63,1.16	0.78,1.82

Figures are odds ratios and 95% confidence intervals from logistic regression. Results are adjusted for complex sample design and include sample weights.

*p<0.05; ** p<0.01; ***p<0.001.

**Figure 1:**
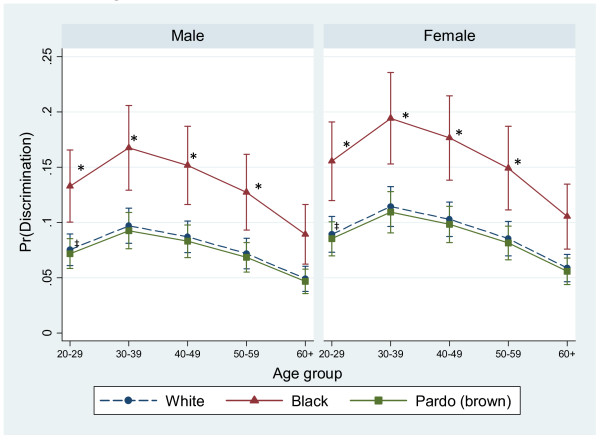
**Predicted probability of reporting any discrimination by age group, skin color, and gender.** Predicted probabilities of perceived discrimination from logistic regression controlling for variables presented in the figure in addition to schooling, health problems, social status, social trust, and BH city residence, all held at their mean. * Difference is statistically significant from reference category (‡) at the p<0.05 level.

Figure 1 shows how the predicted probabilities for reporting discrimination vary by skin color, age group, and sex, once all other variables are set at their mean. It shows that for every age group, people who identify as black are about twice as likely to report experiencing discrimination and that there is little difference between reports among those identified as white or pardo. There is a nonlinear relationship with age, where probabilities peak at age 30–39, then decline thereafter. The error bars show that the predicted probability of reporting some form of discrimination among people who identify as black (with the exception of those age 60+) is significantly higher than those in the white or brown groups (p<0.05). This relationship is similar for each sex, albeit more pronounced for women. The highest predicted values of perceived discrimination were among black women aged 30–39 (19%) and 40–49 years (17.5%).

**Figure 2:**
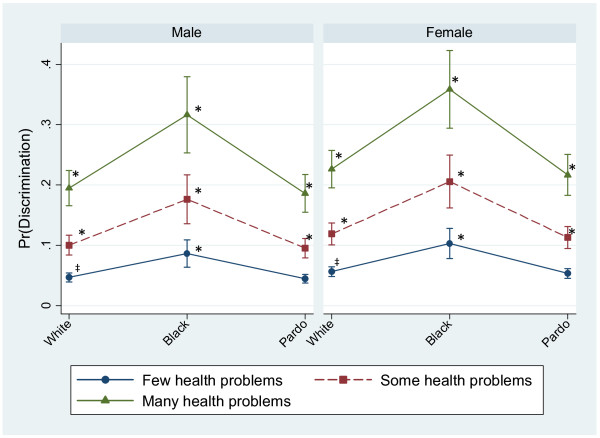
**Predicted probability of reporting any discrimination by health problem, skin color, and gender.** Predicted probabilities of perceived discrimination from logistic regression controlling for variables presented in the figure in addition to age, schooling, social status, social trust, and BH city residence, all held at their mean. * Difference is statistically significant from reference category (‡) at the p<0.05 level.

Figure 2 illustrates relationships among skin color, gender, and health problems. It shows that discrimination reports are highest among those who identify as black, those who report many health problems, and higher for women as compared to men. These differences are statistically significant (p<0.05) when compared to the references group (people who identify as white and have few health problems) among both men and women. The highest predicted probabilities of perceived discrimination were among black men and women in poor health (31% and 35%, respectively).

## Discussion

This study found that in a large metropolitan area, discrimination is strongly associated with a number of individual and contextual characteristics, including skin color, gender, age, and social position. An important finding is the strong and consistent association between being in poor health status and experiencing discrimination. Place also matters. Residence in Belo Horizonte was protective for whites, but was a risk factor for people identifying as black.

The prevalence of overall discrimination (8.6%) was slightly lower than reported in other studies, which varied from 12% among residents of São Paulo to 13% in a nationally-representative opinion poll from 2003 [19,38]. Some of this difference may be due to the fact that each study posed questions about discrimination differently and may also represent regional differences since our study took place in a single metropolitan area.

Reports of discrimination in healthcare settings in this study were within the low end of the range reported elsewhere, which ranged from about 1% (based on skin color) to 13% (based on economic status) of participants who received outpatient and inpatient care, respectively.[20] In studies of other national health systems, experience of perceived prior discrimination in healthcare settings may lead to subsequent underuse of some types of healthcare services, and may also alter the way medical advice is interpreted and acted upon [3]. In our study, multivariable models show that identification as “black” was associated with higher odds of reported discrimination in all settings, except for discrimination experienced in healthcare settings—a finding consistent with other reports [22]. However, women and the poor were more likely to report having experienced discrimination in seeking healthcare—a finding that may have implications for the way the Brazilian health system tackles its ongoing process of quality improvement and “humanization”, that is, making the national health service more accommodating and appropriate for its users [39].

The nonlinear association between age and discrimination requires further investigation. One explanation may be that different age cohorts experienced or perceived discrimination in different ways, or differ in their attitudes towards reporting it. It is also possible that by controlling for health problems in our models, we are capturing more directly some of the reasons why people in the older age groups may experience discrimination-perhaps due to mobility limitations or other reasons associated with poor health. This interpretation is supported by the reduction of standard errors for the age 60 and over group observed before (model 5) and after (model 6) introduction of the health measures.

Our study found that people in poor health have consistently higher odds (about 5 times higher than those with very few health problems) of reporting discrimination. However, the association between poor health and discrimination while seeking medical care was not found to be any higher than in other types of settings. We expected this relationship to be more pronounced, given that people with more health problems should have had increased contact with the health system. Although we cannot establish causal relationships linking discrimination and poorer health, there is considerable evidence on this association. However, longitudinal studies in the Brazilian context are scarce [19,23,40-42]. More nuanced measures of discrimination in a longitudinal context will be necessary to tease out the complex causal relationships between discrimination and poor health in Brazil.

The finding of higher experiences of discrimination among people identifying as “black” is also consistent with other studies [19,21]. However, the implications of this finding need to be understood within the Brazilian context. First, as in other countries, ways of measuring and defining categories of skin color and the meanings assigned to these categories are complex social constructions. In Brazil, there is considerable evidence of how changing social attitudes towards skin color have led to increased social desirability for individuals to identify as lighter skin color [43]. At the same time, some social movements have called for wider acceptance of “black” identity and African heritage. The implication for interpreting findings from this study is that people who chose to classify themselves as “black” may be different from people with otherwise similar features who choose to categorize themselves as “brown”, for example. Recent studies have shown that Brazilians tend to self-classify their skin color near the center of the color gradient, indicating a preference for the “brown” category and its association with the idea of a “mixed-race nation” [22]. The distribution of individuals who self-classified as black along the color scale may indicate either that the value attributed to their skin color is somehow different and/or that “black” represents a political identity adopted by some in order to indicate African ancestry and not merely the color of their skin [22]. The literature suggests that heightened racial or ethnic identification may either protect (due to decreased internalization of negative stereotypes) [44] or exacerbate (through greater vigilance and therefore enhanced awareness of discriminatory acts) the link between discrimination and health [12].

Skin color in Brazil, as in other countries, is also correlated with social class, education, income, and geography [16,45-48]. Consequently, Brazilian studies linking skin color with health outcomes have shown mixed results. In one survey, social inequalities in self-rated health and in the use of health services were not associated with race/skin color once education and economic status were controlled [22]. In another study, the association between discrimination based on skin color and poor health status was significant even after controlling for these factors [23].

The finding that traditional “objective” measures of socioeconomic status (household wealth and education) were not associated with reports of discrimination is unique. Part of this relationship may be explained by the presence of other important demographic variables (age and sex) as well as skin color in multivariable models. This study also points to the potential usefulness of subjective social status (via the MacArthur ladder) as an alternative measure of social position. Studies have shown it to be an important complement to other measures of SES [11] although to our knowledge, this is the first time it has been used in a large household health survey in Brazil.

Social trust was another consistent predictor of discrimination. There is evidence that social support may moderate the effects of discrimination on health, [49] although from this cross-sectional study we do not know if people have low trust due to past experiences of discrimination or whether people who have less trust are more likely to interpret others’ actions as discriminatory or to report acts of discrimination more frequently.

Finally, residence in the city of Belo Horizonte was found to be protective for whites, but a risk factor for people identifying as black. Explanations for this relationship could be due to population density (greater exposure to people may increase odds of experiencing discrimination), but if this were the case we would have expected to have found a significant interaction between urban residence and gender (which we did not). Other explanations such as racial residential segregation (and important explanatory factor in studies conducted in the United States) also appear to be inadequate to explain this finding, since such concentration in Brazil is more apparent at the state and regional levels than within cities--at least within the Belo Horizonte metropolitan region [50]. The result could also be an artifact of the meaning assigned to identifying as “black”, among residents in Belo Horizonte city--the largest metropolitan area in the region.

This study has several strengths and weaknesses. Perceived discrimination should have high external validity because it represents the lived experience of individuals, but it may also present an incomplete picture of reality [51]. There is some evidence that individuals may fail to acknowledge acts of discrimination in order to avoid feeling that they do not have control over situations, or may prefer not to recall or to report such situations [52,53]. Consequently, in this study and others, it is safe to assume that many experiences of discrimination have been underreported.

More important may be limitations on our ability to interpret the motive behind the discrimination that respondents report. We do not know if these are isolated experiences or long-term processes. We also cannot tease out the actual motivation for the discriminatory act, although, there does not appear to be evidence that any one type of discrimination is necessarily more severe in its health effects than any other type [12]. Further Brazilian studies should test more specific measures of discrimination in order to better characterize the relationship between different types and motivations for unfair treatment and their potentially differential effects on health and well-being [25].

In conclusion, we have found that experiences of discrimination are prevalent in a large urban metropolitan region in a multiracial society that has wide-reaching social inequalities. While skin color was an important predictor of discrimination, other characteristics, such as poor health, gender, social status, and trust were also important and powerful risk factors. Our study suggests that discussions in Brazil about discrimination—both within and outside the context of the health system-should not be limited only to questions about race. Although racism is clearly an issue in Brazil and elsewhere, this study supports recent calls to address a wider set of interrelated factors within and across different population groups [39].

## Competing interests

The authors declare that they have no competing interests.

## Authors’ contributions

JM and MFLC conceived the study. JM carried out data analysis and JM and PM drafted the text. All authors helped to draft and revise the manuscript and interpreted results. All authors read and approved the final manuscript.
